# Veterinary Medical Association of New York County

**Published:** 1897-12

**Authors:** Robert W. Ellis

**Affiliations:** Secretary


### VETERINARY MEDICAL ASSOCIATION OF NEW YORK COUNTY.

THE regular monthly meeting was called to order at 8.30 P.M., Wednesday, November 3, 1897, at the New York Academy of Medicine, with the Vice-President, Dr. Robertson, in the chair. The following members responded to roll-call: Drs. Amling, C. C. Cattanach, J. S. Cattanach, J. S. Cattanach, Jr., Delaney, Ellis, Gill, Hanson, Lamkin, MacKellar, Neher, Robertson, Ryder, and Winslow (14). The minutes of the previous meeting were read and approved. The *Comitia Minora* having no report to make, reading of papers was next in order.

Dr. Neher gave the meeting the benefit of his experience in "double current irrigation" and exhibited several instruments employed by him for that purpose, and also gave an interesting little "talk" on the indications for its use. After a free discussion on this subject, Dr. Lamkin opened a discussion on the diagnostic value of mallein, stating that he did not feel disposed to rely upon it, and reported several cases to substantiate his views; the discussion on this subject soon became general, and lasted for a considerable time.

Moved by Dr. Hanson, and seconded by Dr. Gill, that a vote of thanks be extended to Dr. Neher for his "talk" on "double current irrigation" and exhibition of the instruments, and to Dr. Lamkin for his report on the use of mallein; carried.

Dr. Gill also made the suggestion that Dr. Neher send a cut of the instrument to the veterinary journals for the benefit of the profession.

*Reports of Special Committees.* Publication: Dr. Gill reported for this committee that all the matter connected with New York was complete, and that the rest of the matter was in such shape as to be out in about two weeks. Moved and seconded that the report be accepted; carried.

Moved by Dr. Hanson and seconded by C. C. Cattanach, that the Secretary notify all members of their indebtedness to the Association before the next meeting; carried.

[1] See p. 779.

Moved by Dr. Gill that a committee be appointed to investigate in reference to registration of Messrs. Grenside and Hingston; seconded; carried.

President Robertson appointed Dr. Gill to act as a committee of one to search the register. Moved and seconded that the meeting adjourn; carried.

ROBERT W. ELLIS,
Secretary.

